# Detection Method of Citrus Psyllids With Field High-Definition Camera Based on Improved Cascade Region-Based Convolution Neural Networks

**DOI:** 10.3389/fpls.2021.816272

**Published:** 2022-01-24

**Authors:** Fen Dai, Fengcheng Wang, Dongzi Yang, Shaoming Lin, Xin Chen, Yubin Lan, Xiaoling Deng

**Affiliations:** ^1^College of Electronic Engineering, College of Artificial Intelligence, South China Agricultural University, Guangzhou, China; ^2^National Center for International Collaboration Research on Precision Agricultural Aviation Pesticide Spraying Technology, Guangzhou, China; ^3^Guangdong Laboratory for Lingnan Modern Agriculture, Guangzhou, China; ^4^Guangdong Engineering Technology Research Center of Smart Agriculture, Guangzhou, China

**Keywords:** citrus psyllids, deep learning, small target detection, cascade R-CNN, small target enhancement

## Abstract

Citrus psyllid is the only insect vector of citrus Huanglongbing (HLB), which is the most destructive disease in the citrus industry. There is no effective treatment for HLB, so detecting citrus psyllids as soon as possible is the key prevention measure for citrus HLB. It is time-consuming and laborious to search for citrus psyllids through artificial patrol, which is inconvenient for the management of citrus orchards. With the development of artificial intelligence technology, a computer vision method instead of the artificial patrol can be adopted for orchard management to reduce the cost and time. The citrus psyllid is small in shape and gray in color, similar to the stem, stump, and withered part of the leaves, leading to difficulty for the traditional target detection algorithm to achieve a good recognition effect. In this work, in order to make the model have good generalization ability under outdoor light condition, a high-definition camera to collect data set of citrus psyllids and citrus fruit flies under natural light condition was used, a method to increase the number of small target pests in citrus based on semantic segmentation algorithm was proposed, and the cascade region-based convolution neural networks (R-CNN) (convolutional neural network) algorithm was improved to enhance the recognition effect of small target pests using multiscale training, combining CBAM attention mechanism with high-resolution feature retention network high-resoultion network (HRNet) as feature extraction network, adding sawtooth atrous spatial pyramid pooling (ASPP) structure to fully extract high-resolution features from different scales, and adding feature pyramid networks (FPN) structure for feature fusion at different scales. To mine difficult samples more deeply, an online hard sample mining strategy was adopted in the process of model sampling. The results show that the improved cascade R-CNN algorithm after training has an average recognition accuracy of 88.78% for citrus psyllids. Compared with VGG16, ResNet50, and other common networks, the improved small target recognition algorithm obtains the highest recognition performance. Experimental results also show that the improved cascade R-CNN algorithm not only performs well in citrus psylla identification but also in other small targets such as citrus fruit flies, which makes it possible and feasible to detect small target pests with a field high-definition camera.

## Introduction

The prevention and control of agricultural pests and diseases is a very serious problem in agriculture. Farmers usually need to spray a lot of pesticides to prevent pests and diseases in advance. If the field pests can be detected as early as possible, the pesticides can be accurately controlled and reduced. Citrus Huanglongbing (HLB) is one of the most serious diseases that endanger the development of the world’s citrus industry. It has caused a huge blow to the citrus industry in China, the United States, Brazil, Mexico, South Africa, and South Asia. The citrus psyllid is the only insect vector of citrus HLB, and it reproduces fast, has a strong ability to transmit the virus by sucking sap, and is difficult to identify because of its small size (average size of 2.5 mm), so early detecting of citrus psyllids and controlling their transmission are the key measures for prevention and control of HLB (Dala-Paula et al., 2019; Han et al., 2021; Ngugi et al., 2021). Citrus psyllids need an adapted host, mainly shoots, to survive (Gallinger and Gross, 2018). Traditional agricultural measures mainly kill citrus psyllids regularly with pesticides, which lead to the problems such as waste of agricultural materials and environmental and fruit pollution. There is 70–80% chance that citrus psyllids will transmit the HLB pathogen to healthy trees when they feed on the sap from the leaves of HLB trees and then fly to healthy trees. If farmers can detect citrus psyllids as soon as possible and spray pesticides accurately, the number of psyllids can be effectively reduced, the probability of psyllids sucking HLB diseased trees can be greatly reduced, and the transmission of HLB through psyllids can be effectively controlled. Therefore, through early detection and early control method, the population of citrus psyllids can be reduced, and the spread of HLB can be effectively prevented, thereby increasing the yield of citrus.

With the development of deep learning technology and the improvement of hardware equipment, the feasibility of image recognition of diseases and pests is constantly improving, more and more algorithms have been applied to the detection of plant diseases and pests (Ngugi et al., 2021). Accumulating evidence highlights the potential of employing CNNs in plant phenotyping settings. Their incorporation was proven to be very effective due to their capacity of distinguishing patterns and subtracting regularities from information under analysis. In plant sciences, there are many relevant and successful implementations including identification by examining seeds (chickpea; Taheri-Garavand et al., 2021a) or leaves (grapevine; Nasiri et al., 2021), tomato pest detection based on improved YOLOv3 (Liu and Wang, 2020, detection of mango anthracnose using neural networks (Singh et al., 2019), recognition of disease spots on soybean, citrus, and other plant leaves by deep learning (Arnal Barbedo, 2019), identification of rice-diseased leaves using transfer learning (Chen J. et al., 2020), classification and identification of agricultural pests in complex environments (Cheng et al., 2017), real-time detection of apple leaf diseases and insect pests (Jiang et al., 2019). These studies have shown that the neural network is successfully modeled under laboratory or field conditions with good recognition effect even under complex conditions, and transfer learning can also be performed according to different objects.

Convolutional neural network for image classification has become a standard structure to solve visual recognition problems, such as ResNet (He et al., 2016), VGGNet (Simonyan and Zisserman, 2014), GoogLeNet (Szegedy et al., 2014), and ResNetXt (Xie et al., 2016). The characteristic of these networks is that the learned representation gradually decreases in spatial resolution, which is not suitable for regional and pixel-level problems. The features learned through the above classification network essentially have low-resolution features. Therefore, the huge loss of resolution makes it difficult for the network to obtain accurate prediction results in tasks that are sensitive to spatial accuracy.

Target detection is constructed to solve the problems of classification and regression. At present, the target detection models based on deep learning are mainly divided into two categories: the two-stage method represented by faster region-based convolution neural networks (R-CNN) (Ren et al., 2017) and the one-stage method represented by Single Shot MultiBox Detector (SSD) (Liu et al., 2016). Although many different target detection algorithms have emerged, such as faster R-CNN (Ren et al., 2017), YOLO (Redmon et al., 2016), and other target detection algorithms, which achieve high recognition accuracy on conventional objects such as pedestrians and vehicles. However, the target of agricultural pests such as citrus psyllids is too small to be recognized by the above target detection algorithms. Chen et al. (2016) defined small targets with the characteristics of low-pixel occupancy in the whole picture, small candidate box, insufficient data sample, and so on. Because of these characteristics, the algorithms for small goals are still stuck in specific occasions, for example, building recognition in high altitude remote-sensing images (Xia et al., 2017), recognition of traffic lights in pictures (Behrendt et al., 2017), pedestrian recognition from the driver’s perspective (Zhang et al., 2017).

The citrus psyllid studied in this experiment has the characteristics of small size, gray color, and easily being mistaken as branches, stems, and dead leaves. The deeper the layers of the neural network are, the more information will be lost. Therefore, it is difficult to extract useful feature information from the network for small target citrus psyllids. The similarity in color makes it difficult to identify the target, which makes citrus psyllids often be considered as branches or dead leaves. Besides, the distribution of citrus psyllids is scattered, often concentrated in the bud, leaf back, and leaf veins, so each picture does not necessarily have a large number of psyllid samples. For images with fewer samples, the number of trainable positive sample boxes is greatly reduced, and it is not easy to train a model with superior performance. Cascade R-CNN (Cai and Vasconcelos, 2017) is a two-stage target detection model framework proposed in recent years, solving the IoU selection problem of the traditional target detection algorithm by cascading various detection models and having good detection performance for small targets. Therefore, to solve the problem of lack of citrus psyllids, a method of citrus psyllids enhancement based on semantic segmentation was explored, and the cascade R-CNN model for the small target recognition of citrus psyllids was improved in this study.

## Materials and Methods

### Data Acquisition and Processing

**FIGURE 1:**
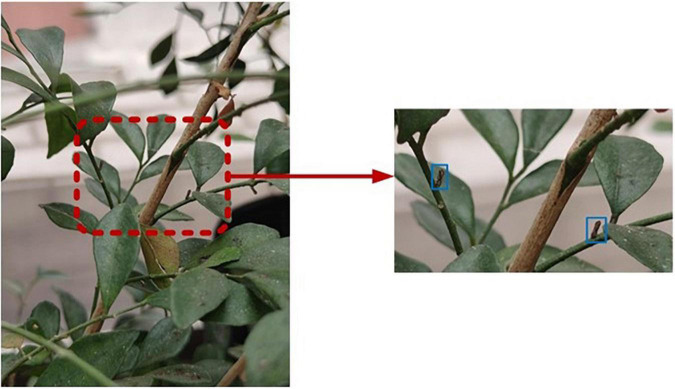
Experimental data. The blue box is used to mark citrus psyllids.

The main location for collecting the data in this study is the Citrus HLB Test Base of South China Agricultural University (Longitude: 113.35875, Latitude: 23.15747), in Guangdong Province, China. The data collected in this experiment mainly used RGB images (visible spectrum 400–700 nm). The collected instruments include a mobile phone (Huawei Mate 40, China) with high-definition cameras and a Sony camera (Sony, ILCE-6400, made in China), with 4,000 × 5,000 pixels. The shooting distance was controlled within the range of 50–100 cm, and the shooting angle and orientation were not fixed. The shooting was performed in the morning, noon, and afternoon on a sunny day and under normal lighting condition. The targets in the picture are mainly citrus psyllids (average size of 2.5 mm) and fruit flies (average size of 5 mm) which are the main pests in citrus orchards. Although the citrus fruit fly is larger than the citrus psyllid, it still has the characteristics of being small and difficult to detect. In this study, the data of citrus psyllids and citrus fruit flies were used for model training. The model is proved to be transplantable to other small target pests by adding citrus fruit flies to the training. The data were collected in the spring of March, April, and May, from different Rutaceae plants (*Rutaceae Juss*.) including Shatangju (*Citrus reticulata Blanco*), kumquat potted plant [*Fortunella margarita* (Lour.) Swingle], and *Murraya exotica* (*Murraya exotica L.*) potted plant. Finally, a total of 500 high-definition sample images were obtained. The expensive price experimental data are shown in Figure 1.

### Target Sample and Data Set Enhancement

**FIGURE 2:**
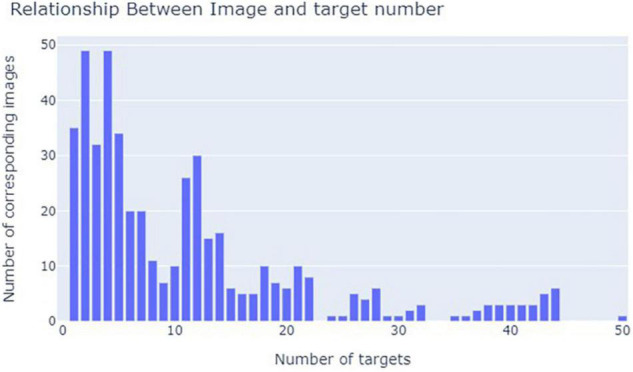
Diagram of the relationship between the picture and the target quantity.

The relationship between the number of targets and images was analyzed through observation and mathematical statistics and is shown in Figure 2. Most of the pictures contain a small number of psyllid samples. The size of the citrus psyllid is much smaller than the size of the entire image, so this research belongs to the small target detection range.

The number of citrus psyllids in the picture can influence the training effect of the model. The more the number of psyllids in the picture, the more positive samples will be produced during the training, and the more the model will learn the characteristic information of psyllids. Therefore, to improve the identification effect of psyllids, the first step is to increase the sample number of citrus psyllids in the picture. For this kind of small target, there are many ways to enhance the small target, such as component stitching (Chen Y. et al., 2020), artificial augmentation by copy-pasting the small objects (Kisantal et al., 2019), AdaResampling (Ghiasi et al., 2020), and scale match (Yu et al., 2020). Due to the randomness of the target distribution, the number of targets distributed in each image is inconsistent. After cropping, a lot of pictures lack psyllid samples, which causes great difficulties in the recognition of the neural network.

**FIGURE 3:**
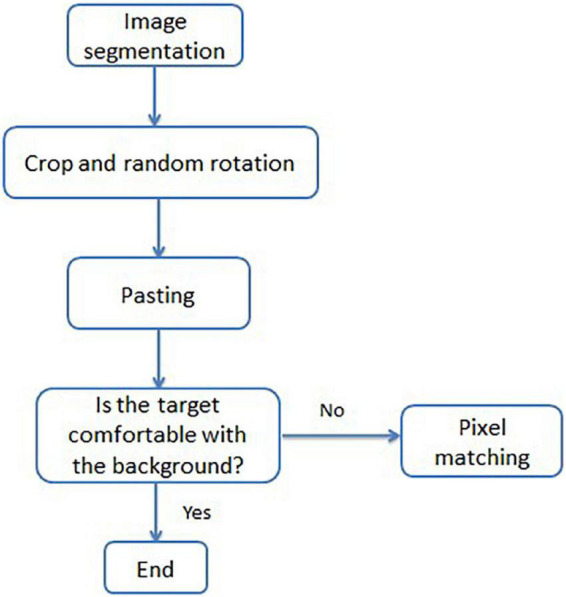
Small sample number enhancement flowchart.

To enhance the training effect of the model and improve the overall generalization ability of the model, the diversity of small target positions was increased by copying and pasting small targets multiple times and randomly pasting targets that do not overlap with existing targets. Based on the principle of replication, a method to increase the number of citrus psyllids was proposed, and the process is shown in Figure 3.

First, the pretrained semantic segmentation model U-Net (Ronneberger et al., 2015) was adopted to remove the redundant background of each image in the training set, only retaining the leaves and tree trunks. Then, each citrus psyllid sample was randomly copied and pasted onto the leaf or trunk position, ensuring that the pasted position does not coincide with the current position. Multiple copy and paste operations on different pictures with fewer targets were performed. The pasted target may not be in harmony with the background of the pasted position. When the pasted target is too bright or too dark compared with the surrounding background, the neural network will be very sensitive to the difference. The trained model can only have good generalization ability for the enhanced image, but a poor detection effect for the unenhanced natural lens image. Therefore, the color of the target that cannot be integrated into the background after pasting was modified manually, so that the target is as harmonious as possible with the background of the pasting place. The calculation formula for the overlap rate of the outer region (the background part removed by segmentation) and the sample overlap rate of the inner region (the leaves and trunk parts) is defined as Eqs. 1 and 2, where *U_Outer region_* is the overlap rate of the outer region and *U_Samples_* is the sample overlap rate of the inner region; *Area_copy_* represents the area where the sample is located after copying, *Area_outer region_* represents the outside area, and *U_Outer region_* represents the degree of overlap between the area where the sample is located and the outer area after copying. The larger the *U_Outer region_*, the larger the area where the sample is located in the outer area after copying; n is the total number of original image samples; *Area_Samples_* represents the total area occupied by the original image samples, and *U_Samples_* represents the degree of overlap between the area occupied by the copied samples and the area occupied by the original image samples.


(1) $$U_{\mathrm{Outer}\mathrm{region}}=\frac{{\mathrm{Area}}_{\mathrm{copy}}}{{\mathrm{Area}}_{\mathrm{outer}\mathrm{region}}}$$

(2) $$U_{\mathrm{Samples}}=\frac{{\mathrm{Area}}_{\mathrm{copy}}}{{\mathrm{Area}}_{\mathrm{Samples}}}(Samples=Sample1+Sample2+\ldots +SampleN)$$

During the enhancement process, the *Area_copy_* of the sample area after being selected and pasted needs to meet the following conditions:


(3) $$U_{\mathrm{Outer}\mathrm{region}}=0,U_{\mathrm{Samples}}=0$$

**FIGURE 4:**
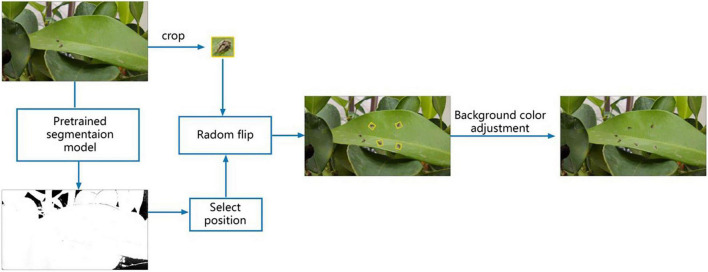
Small sample number enhancement example diagram.

That is, the copied sample area needs to be in the area without overlapping with the original sample area. The example process of data enhancement is shown in Figure 4.

The white part in the segmentation diagram is the inner area, and the black part is the outer area.

Furthermore, the performance of the model is affected by the number of training sets. With the increase in training data, the recognition performance of the model will be improved to a certain extent. To increase the training data set and improve the general recognition ability of psyllids, offline resampling was used in the experiment. Two times the resampling rate was used to process the image set after the target samples were enhanced in the image. Since the image needs to be preprocessed before being input to the network, the data are not exactly the same after preprocessing, so there will be no overfitting of the training set.

### Image Preprocessing

**FIGURE 5:**
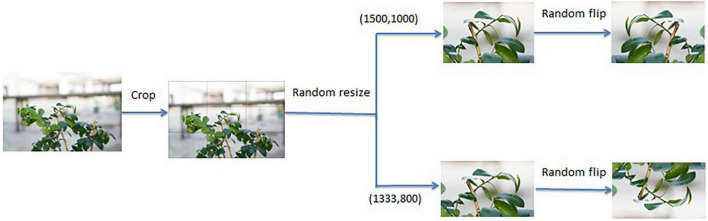
Image cropping flowchart.

The image set captured in this study was high-resolution (4,000 × 5,000 pixels). If these data are directly input into the network for training, it will lose a lot of useful citrus psyllid information due to compression during the training process. Therefore, the high-resolution images were cropped into nine blocks before being input into the network in this study. The size of the input image affects the performance of the detection model, and the feature map generated by the feature extraction network is often dozens of times smaller than the original image, which will make it difficult for the detection network to capture the feature description of citrus psyllids. Therefore, this study uses multiscale training to improve the performance of the model. In view of the advantages of multiscale training, two scales (1500, 1000) and (1333, 800) were set, and each scale was randomly selected for training in each epoch. To increase the diversity of training samples, the input image was rotated randomly with 50% probability, and the image cropping flowchart is shown in Figure 5.

### Improved High-Resoultion Network for Feature Extraction

High-resoultion network (HRNet) (Sun et al., 2019) can learn enough high-resolution representations, which is different from the traditional classification network. In view of the small size of the citrus psyllids, if the traditional neural network is used for feature extraction, the key feature information of the shape and color of the citrus psyllids or the high-resolution feature information can be easily lost in the last layer of the network, whereas HRNet can maintain the high-resolution representation of citrus psyllid features by connecting high-resolution and low-resolution convolutions in parallel and enhance the high-resolution representation of citrus psyllid features by repeatedly performing multiscale fusion across parallel convolutions. It can achieve better results in small-area classification such as citrus psyllids. Therefore, HRNet was adopted as a feature extraction network to reduce the information loss of citrus psyllid features in this study.

**FIGURE 6:**
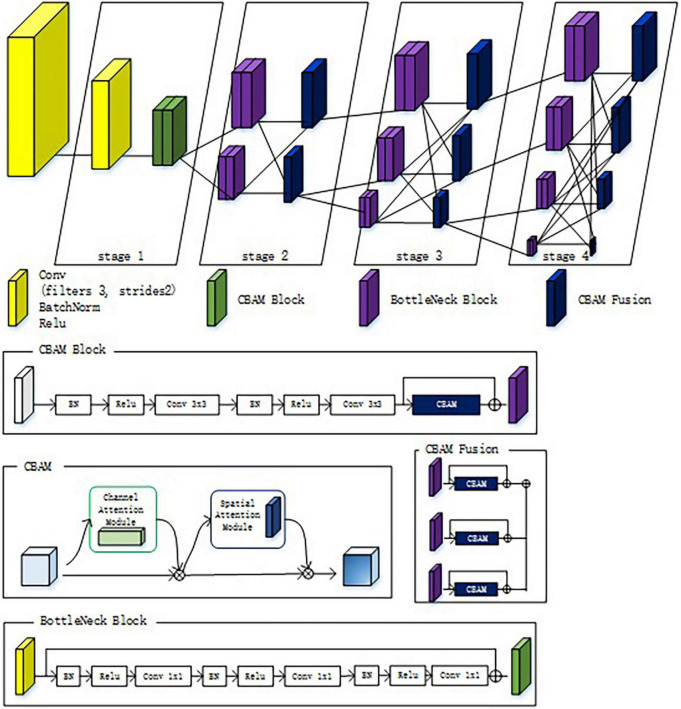
High-resoultion network (HRNet) model with CBAM attention mechanism.

To make the network pay more attention to the characteristics of citrus psyllids, a lightweight attention mechanism convolution block attention module (CBAM) (Woo et al., 2018) was added to the network, which is an attention mechanism module combining space and channel, where channel attention mechanism focuses on what features are meaningful from the perspective of channel, while space attention mechanism focuses on what features are meaningful from the space scale of image. In this study, the CBAM attention mechanism was added to the first-stage feature extraction and the second-, third-, and fourth-stage feature fusion of HRNet as shown in Figure 6. Adding CBAM blocks to the first stage enables the network to lock the target features that need attention in the initial stage of feature extraction. Adding the CBAM fusion module to the second stage, the third stage, and the fourth stage fully enable the extraction of the important citrus psyllid feature information of different resolutions when fusing the features of different resolutions.

### Feature Fusion Strategy

**FIGURE 7:**
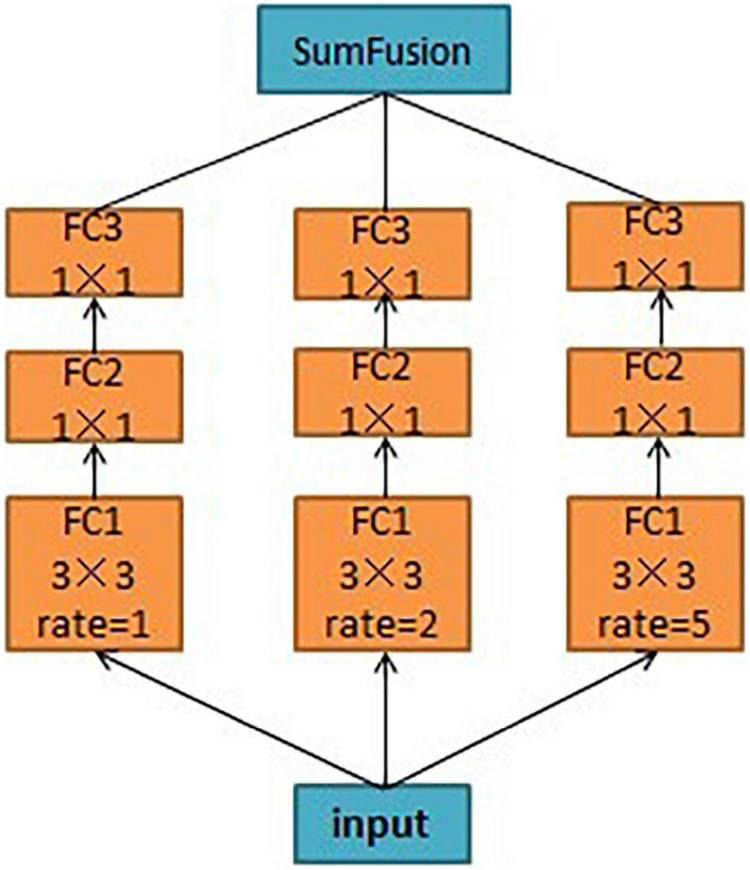
Atrous spatial pyramid pooling (ASPP) structure.

**FIGURE 8:**
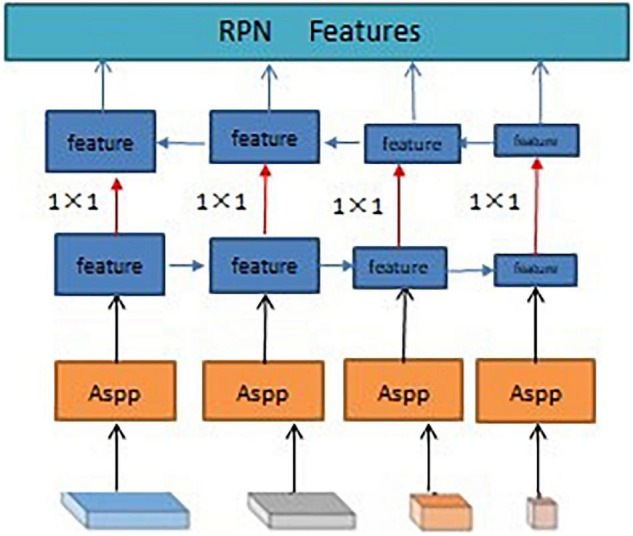
Feature pyramid networks (FPN) structure.

As shown in Figure 6, the final network layer of the HRNet outputs four feature maps with different resolutions. Because of the small size of the citrus psyllids, the information of the citrus psyllid characteristic map at each resolution may be lacking, and different resolutions’ information needs to be combined to complement each other. To make full use of the feature maps at different resolutions, the atrous spatial pyramid pooling (ASPP) (Chen et al., 2017) (dilated space convolution pooled pyramid) structure was used for feature fusion at different resolutions in this study. In ASPP, the extracted characteristics of citrus psyllids are input into the dilated convolutions at different sampling rates, which is equivalent to capturing the characteristic information of citrus psyllids at multiscale. The dilated convolutions in the ASPP structure can expand the field of view without losing the resolution, and features under different dilation rates are collected in parallel to obtain the multiscale information of citrus psyllids, and such these operations can improve the recognition effect of the entire network, which is shown in Figure 7. However, ASPP only uses a large dilation rate [such as (1, 3, 6, 12)] which is only effective for large object detection, but not suitable for citrus psyllid detection. To make full use of the advantages of ASPP and more suitable for the characteristics of small target detection in this study, the dilation rate was designed into a zigzag structure, the dilation rate was set to (1, 2, 5), and the feature pyramid networks (FPN) (Pan et al., 2019) which is shown in Figure 8 was used to fuse the 4 citrus psyllids features at different resolutions processed by ASPP.

Besides, the shape and color of citrus psyllids are similar to branches, tree stems, and dead leaves, so the model will produce more positive and negative samples with higher loss during the training process. For example, the model predicts part of the trunk as citrus psyllids. Using the traditional random sampling method, a large number of difficult samples, such as branches, may be missed. The model trained using the random sampling method cannot distinguish citrus psyllids, tree branch, and tree stem. So, in this study, random sampling method in cascade R-CNN was replaced by an online hard sample mining strategy (Shrivastava et al., 2016), and the suggestion box with high-loss value was given high priority to be sampled. The strategy is as follows: in the training process, the ROI loss in each stage is sorted, and the first 64 samples of ROI loss in the positive samples and the first 192 samples of ROI loss in the negative samples as training samples are selected according to the sorting structure.

## Results

### Experiment Setting and Evaluation Index

The models for identifying citrus psyllids were trained and tested under the desktop computer with inter-i7-9800x CPU, GeForce GTX 1080ti GPU, Ubuntu 16.04 operating system, and PyTorch deep learning framework. The average detection time of high-resolution images is 10 ms per image. To evaluate the effectiveness of the citrus psyllid detection method proposed in this study, the average precision (*AP*) and mean average precision (*mAP)* were chosen as evaluation indicators, where *AP* is a measure of the average precision value of a category detection, using the precision rate to integrate the recall rate, as shown in eq. (4), *mAP* is a measure of the average value of all types of *AP*, as shown in formula (5).


(4) $$\mathrm{AP}=\int_{0}^{1}\mathrm{Precision}\mathrm{rate}\mathrm{dRecall}\mathrm{rate}$$

(5) $$\mathrm{mAP}=\frac{1}{C}\sum_{c\in C}AP\left(c\right)$$

Where ***c*** represents a certain category, and ***C*** represents the general category.

### Small Target Number Enhancement Results

**FIGURE 9:**
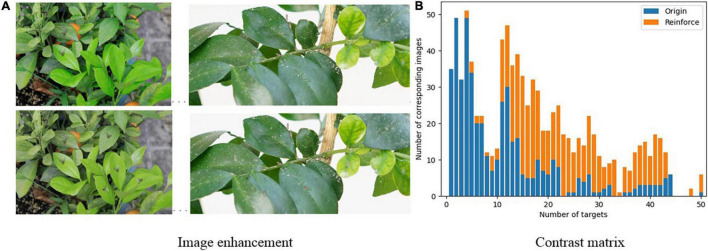
Comparison of the number of small targets before and after enhancement **(A)** image enhancement **(B)** contrast matrix.

Figure 9 shows the comparison matrix of the number of small targets before and after enhancement. The top row of Figure 9A is the original image, and the bottom row is the enhanced image. Figure 9B is the contrast matrix of the number of objects before and after the enhancement. Through observation, it can be found that using the small sample number enhancement method based on the semantic segmentation model improves the number of small targets in each picture in the data set. As the number of small samples per picture increases, a large number of useful positive samples is increased, which can effectively increase the performance of the model.

### Online Hard Sample Mining

**FIGURE 10:**
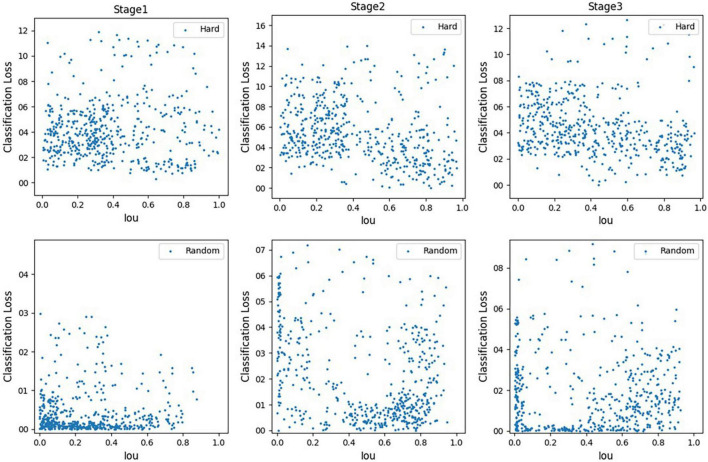
Comparison of difficult sampling and random sampling.

Figure 10 is a comparison diagram of sampling three cascades using random sampling and difficult sample mining methods in cascade R-CNN. It can be found that most random samples are distributed in areas with low classification loss. This is because negative samples contain lots of low-loss samples, so random sampling has a high probability of collecting these low-loss samples. However, the generalization ability of the model trained with lots of low-loss samples is not strong, and it could not classify hard samples well. Also, it shows that the samples collected by the hard sampling method are concentrated in the high-loss area. This is because the hard sample mining method will give priority to the samples with high classification loss, even if the samples contain a large number of samples with low loss. Therefore, the online hard sample mining algorithm can improve the ability of the model to identify difficult samples, so as to improve the overall performance of the model.

### The Performance of Modeling

Some common models that are ResNet, VGGNet, and ResNetXt were adopted for comparison with the proposed method in this study. The *AP* and *mAP* values of the test results of the models are shown in Table 1. Data enhancement represents small target number enhancement and offline resampling. In Table 1, it can find that the improved HRNet + Data enhancement model has 9.0% higher *AP* than the improved HRNet + Offline resampling model. It can also be found that using small target number enhancement can improve the recognition effect of citrus psyllids to some extent. From the comparison, the *AP* values of the HRNet model were 6.14, 7.09, 5.76, and 6.65% higher than those of ResNet50, ResNet101, next101, and VGG16, respectively. The reason is that citrus psyllid is small in size, and it is easy to lose information when using traditional CNN to extract the characteristics of citrus psyllids. The model cannot fully learn the shape, size, distribution, and color information of citrus psyllids, so it does not have good generalization ability. The background area in the picture is much larger than the total area of the citrus psyllids. The model needs to find out the characteristic information of the citrus psyllids from many characteristic information. Therefore, adding an attention mechanism can enhance the attention of the network to the characteristic of the citrus psyllids, extracting key information from the shape, distribution, color, and size of citrus psyllids. In Table 1, the improved HRNet + Data enhancement model has higher *AP* than the HRNet model, which proves that adding the attention mechanism can improve model performance. The comprehensive improvement scheme (improved HRNet + ASPP + FPN + Online hard sample mining strategy + Data enhancement) has the best performance in detecting citrus psyllids, which is more than 10% higher than that of other models. This shows that the addition of ASPP and FPN structures can fully complement the characteristics of citrus psyllids at different resolutions and solve the problem of lack of information on citrus psyllids at a single resolution. Adding an online hard sample mining strategy can allow the model to focus on learning features of objects similar to citrus psyllids and solve the problem of indistinguishable branches, stalks, and dead leaves from citrus psyllids.

**TABLE 1:** Different model recognition effects.

Models	Average precision		Mean average precision
	Citrus psyllids	Fruit flies	
ResNet50 + Data enhancement	67.4%	78.48%	72.94%
ResNet101 + Data enhancement	66.45%	75.33%	70.89%
ResNetXt101 + Data enhancement	67.78%	77.82%	73.3%
VGG16 + Data enhancement	66.89%	77.37%	72.13%
HRNet + Data enhancement	73.54%	80.25%	76.89%
Improved HRNet + Data enhancement	81.89%	84.73%	76.89%
Improved HRNet + Offline resampling	72.89%	76.25%	74.57%
Improved HRNet + ASPP + FPN + Online hard sample mining strategy + Data enhancement	88.78%	91.64%	90.21%

The final proposed model not only performs well in the detection of citrus psyllids but also achieves good recognition performance on small targets such as citrus fruit flies. Citrus fruit flies are larger and have more obvious appearance characteristics than citrus psyllids, such as double wings and heads, so they are easier to identify than citrus psyllids. However, citrus fruit flies are also in the recognition range of small targets, and there are fewer pixels in the identifiable area. Therefore, it is difficult to accurately extract the characteristic information of fruit flies. Through Table 1, it can be found that the final model proposed can achieve 91.64% accuracy in citrus fruit fly recognition.

Table 2 shows the visual prediction results of each model on the original data. The red box is the citrus psyllid label, and the blue box is the prediction result. From the results, it can be found that the model using the improved HRNet as the feature extraction network can achieve a better recognition effect. At the same time, comparing the model of ResNet50 and the model of ResNet101 shows that the deeper the network layer is, the more unfavorable it is to recognize the citrus psyllids. From the results of the heat map, it shows that the model proposed in this study based on improved HRNet + Data enhancement + SPP + FPN + online hard sample mining can better extract the feature information of citrus psyllids.

**TABLE 2:** Visualization results of different models.

Model	Data	Visualization of results	Heat map
ResNet50 + Data enhancement	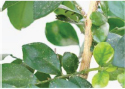	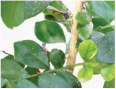	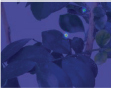
ResNet101 + Data enhancement	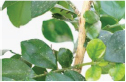	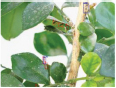	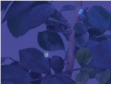
ResNetXt101 + Data enhancement	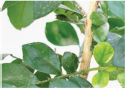	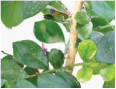	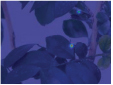
VGG16 + Data enhancement	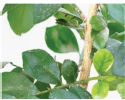	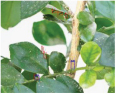	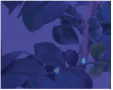
Improved HRNet + Data enhancement	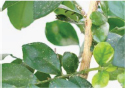	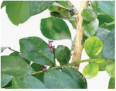	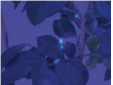
Improved HRNet + ASPP + FPN + Online hard sample mining strategy + Data enhancement	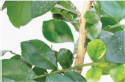	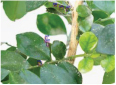	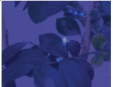

**FIGURE 11:**
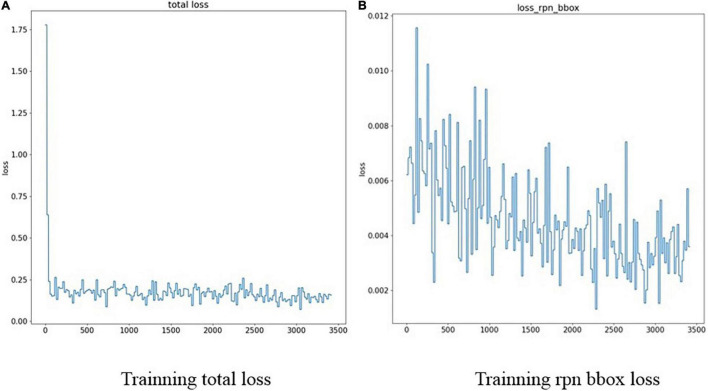
Training loss diagram **(A)** training total loss **(B)** training RPN bbox loss.

Figure 11 shows the training loss diagram of the proposed comprehensive improvement scheme, where Figure 11A represents the total loss curve calculated with 1:0.5:0.25 weights for stage 1, stage 2, and stage 3, and Figure 11B represents the loss curve for RPN bbox on the training set.

**FIGURE 12:**
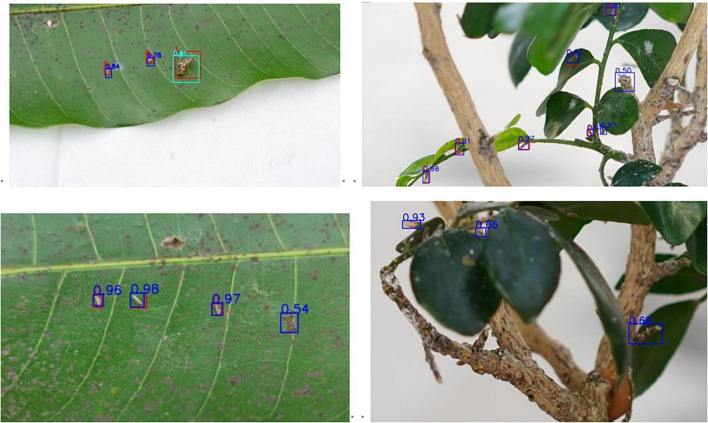
Prediction results.

The prediction results for testing data of the proposed model are shown in Figure 12, where the red box is the label box, the blue box is the citrus psyllids prediction box, and the mint green is the fruit fly prediction box; the label category 0 represents the citrus psyllids and the label category 1 represents the fruit flies. The result shows that the overlap between the label frame and the prediction frame is very high, which proves that the improved model has a good performance in detecting citrus psyllids.

## Discussion

Agricultural pests are small and difficult to be found, and traditional neural networks cannot meet the recognition of small target agricultural pests such as citrus psyllids. This study explores a suitable network and solution for the identification of citrus psyllids. The model proposed in this study can achieve a good recognition effect when the target is larger or slightly smaller than the size of the citrus psyllids, and there are some or no objects similar to the target in the background. Unfortunately, if the detection target is too small, much smaller than the psyllids, such as the red spider citrus (average size of 0.39 mm), the detection effect is not satisfactory using the proposed method of this article. Also, if the detection target is difficult to capture, such as the rice stem borer whose larvae burrow into the rice stalk to eat, it is hard to identify without manual intervention. Besides, if the plant background has a large number of parts that are very similar to the characteristics of the detection target, the recognition accuracy of the method proposed in this article might be reduced.

The use of RGB camera shooting in this study has a good promotion ability without costly equipment and professionals. Outdoor acquisition of RGB images is affected by lighting conditions. When the weather is cloudy, the general brightness of the image will be low due to the lack of light, which will lead to a decrease in the accuracy of target recognition. Besides, the different angles between the camera and the object will affect the recognition accuracy to a certain extent, and these problems can be solved by adding training data sets. If light conditions during acquisition are of interest, there is always the possibility of using scanners (Taheri-Garavand et al., 2021b). In this experiment, the model is trained by inputting pictures with different illumination conditions, distances, and angles. The complexity of the data sources in this experiment shows that this model can be applied to various environments, including laboratory environments and complex external environments, so the model has good generalization ability.

The proposed citrus psyllid detection method based on machine vision can be applied to the actual field monitoring of orchards, and the detection model can be deployed on edge computing devices to help orchard managers more easily monitor the occurrence of orchard pests and summarize the changes. Also, the proposed model can be deployed on terminal devices such as RGB cameras, mobile phones, and cameras mounted on an insect trapper or mobile platform to monitor pests in real time, greatly reducing labor costs, time costs, and resources.

## Conclusion

The citrus psyllid has the characteristics of small size, similar color, and shape to branches, stems, and dead leaves, which causes difficulties to actual field monitoring based on machine vision. To detect citrus psyllids effectively, a comprehensive detection solution based on a high-definition camera for field detection is introduced in this paper. In view of uneven distribution of a small target in the image, a sample enhancement method to increase the number of target samples was first proposed. The detection model was built based on cascade R-CNN, which was improved by using HRNet as the feature extraction network, adding a lightweight attention mechanism CBAM in HRNet to make the network pay more attention to the citrus psyllid features, adding the ASPP structure to extract the high-resolution features from different scales, and integrating the features of different scales with FPN structure. In view of the similarity between citrus and tree branches, tree stems, and dead leaves, online hard sampling mining strategy was adopted. The results show that the improved cascade R-CNN detection model achieved 90.21% *mAP* on the test set, which is much higher than that of other models for the recognition of small targets. After deploying the detection model on edge computing devices, the proposed comprehensive solution can provide real-time detection of citrus psyllids in practical application, reducing the cost of artificial patrol and waste of resources. The solution proposed in this article provides a reference for field camera detection and identification of pests. In addition, early detection and treatment of citrus psyllids can reduce and prevent the occurrence of citrus HLB in citrus orchards.

## Data Availability Statement

The raw data supporting the conclusions of this article will be made available by the authors, without undue reservation.

## Author Contributions

FD conceptualized the experiments, selected the algorithms, collected and analyzed the data, and wrote the manuscript. FW and DY trained the algorithms and collected and analyzed the data. SL and XC wrote the manuscript. XD and YL supervised the project and revised the manuscript. All authors discussed and revised the manuscript.

## Conflict of Interest

The authors declare that the research was conducted in the absence of any commercial or financial relationships that could be construed as a potential conflict of interest.

## Publisher’s Note

All claims expressed in this article are solely those of the authors and do not necessarily represent those of their affiliated organizations, or those of the publisher, the editors and the reviewers. Any product that may be evaluated in this article, or claim that may be made by its manufacturer, is not guaranteed or endorsed by the publisher.

## Abbreviations

HLB: citrus Huanglongbing
CNN: convolutional neural network
CBAM: convolution block attention module.
